# Modulation of Leptin and Serotonin by Honey and Its Glycoproteins Against High-Fat Diet-Induced Metabolic and Anxiety Phenotypes

**DOI:** 10.3390/biomedicines14071641

**Published:** 2026-07-21

**Authors:** Atia Gohar, Richard L. Atkinson, Muhammad Shakeel, Darakhshan J. Haleem, Kaleem Ullah, Aamir Rasool

**Affiliations:** 1 HEJ Research Institute of Chemistry, ICCBS, University of Karachi, Karachi 75270, Pakistan; 2 Dr. Panjwani Center for Molecular Medicine and Drug Research, ICCBS, University of Karachi, Karachi 75270, Pakistan; adv36lab@gmail.com (R.L.A.); shakeel.abh@yahoo.com (M.S.); 3 Division of Endocrinology and Metabolism, Virginia Commonwealth University School of Medicine, Richmond, VA 23103, USA; 4 Jamil-ur-Rahman Center for Genome Research, Dr. Panjwani Center for Molecular Medicine and Drug Research, ICCBS, University of Karachi, Karachi 75270, Pakistan; 5 Department of Microbiology, University of Balochistan, Quetta 87300, Pakistan; drkaleemullah@gmail.com; 6 Institute of Biochemistry, University of Balochistan, Quetta 87300, Pakistan

**Keywords:** serotonin, hyperleptinemia, 5-HIAA, honey proteins, anxiety-like behavior

## Abstract

**Background**: Obesity-linked anxiety may involve leptin resistance, impairing serotonin (5-hydroxytryptamine; 5-HT) signaling. In addition to drugs, nutraceuticals have been explored for their potential to address this pathophysiology. Herein, natural honey and its glycoproteins were assessed for their roles in modulating the leptin–serotonin axis to alleviate metabolic and anxiety-related disturbances. **Methodology**: Sixty Wistar rats were allocated to two groups (*n* = 30 each), one on a normal diet (ND) and the other on a high-fat diet (HFD), for four weeks to induce obesity. Each group was further divided into five sub-groups (*n* = 6 each) as follows: saline, low honey dose (LHD, 1 g/kg), high honey dose (HHD, 2 g/kg), low protein dose (LPD, 0.25 mg/kg), and high protein dose (HPD, 0.5 mg/kg), while continuing on the ND or HFD. The interventions were administered for four weeks. Body weight and behavioral activities were measured weekly, whereas serum leptin and triglycerides, and brain 5-HT and its primary metabolite, 5-hydroxyindoleacetic acid (5-HIAA), were measured at sacrifice. Statistical analyses were performed using ANOVA, Tukey’s post hoc test, and correlation analyses. **Results**: The HFD induced leptin-driven metabolic dysfunction and serotonergic disruption, exacerbating anxiety-like behavior and locomotor deficits compared with the ND rats (*p* < 0.05). HHD produced a dual metabolic–neurochemical improvement by reducing hyperleptinemia, elevating 5-HT, and restoring locomotor activity while reducing anxiety-like behavior. In contrast, HPD increased 5-HIAA without altering 5-HT, reflecting an imbalanced serotonin turnover that yielded only partial anxiolytic effects. Overall, HHD partially reversed HFD-induced pathology through leptin–serotonin crosstalk, whereas HPD provided more targeted neurochemical modulation. **Conclusions**: Natural honey and its glycoproteins attenuate HFD-induced metabolic dysregulation and anxiety-like behavior by counteracting hyperleptinemia and enhancing serotonin synthesis. These findings highlight the potential of diet-personalized, honey-based interventions for metabolic–CNS comorbidities, warranting validation through clinical trials.

## 1. Introduction

Serotonin (5-hydroxytryptamine; 5-HT) is a neurotransmitter synthesized from the amino acid tryptophan by the rate-limiting enzyme tryptophan hydroxylase (TPH). Serotonin is subsequently metabolized into 5-hydroxyindoleacetic acid (5-HIAA) via a two-step reaction involving monoamine oxidase A (MAO-A) and aldehyde dehydrogenase (ALDH) [1]. Serotonin is involved in several physiological processes; notably, it modulates food intake through two distinct central pathways: (i) the homeostatic circuitry, which suppresses consumption upon energy sufficiency, and (ii) the hedonic pathway, which attenuates reward-motivated feeding behavior [2]. Dysregulation of this intricate system, mediated by heterogeneous serotonin receptor populations and their distributed neural connections, has been implicated in the pathogenesis of obesity [3]. Notably, human obesity is characterized by attenuated central serotonergic tone, which may promote hyperphagia through impaired homeostatic inhibition [4]. Although pharmacological targeting of specific serotonin receptor subtypes has demonstrated modulatory effects on feeding behavior and adiposity, clinical translation has been constrained by adverse effects [2,5,6].

The increasing global prevalence of obesity and anxiety, two pathophysiologically co-occurring conditions, highlights the limitations of conventional serotonergic pharmacotherapies [7,8]. Although serotonin critically modulates both satiety signaling and mood, existing drugs targeting this system demonstrate suboptimal efficacy in treating anxiety, and several serotonergic anti-obesity agents have been withdrawn or restricted due to safety concerns [9,10,11]. This therapeutic impasse necessitates the exploration of alternative targets, such as leptin [12,13]. Produced by adipocytes, leptin crosses the blood–brain barrier to mediate satiety signaling and has shown putative antidepressant properties [14]. However, leptin resistance, characterized by elevated circulating leptin levels alongside central insensitivity, is a hallmark feature of both obesity and anxiety, complicating its therapeutic potential [15,16,17].

Understanding leptin resistance could lead to alternative treatments for obesity, anxiety, and their comorbidity, moving beyond the current over-reliance on serotonergic agents, an approach that overlooks the complex hormonal interplay (including leptin) governing mood and metabolism [9]. Preclinical rodent studies have demonstrated that systemic leptin administration (100 or 500 μg/kg) produced multi-modal therapeutic effects relevant to obesity-associated neurobehavioral dysregulation [18,19,20]. Anxiolytic effects were evidenced by increased open-arm exploration in the elevated plus maze, which positively correlated with circulating leptin concentrations [19,21,22]. Endocrinological analyses showed that leptin elevated serum serotonin while suppressing ghrelin levels, with both effects significantly correlated with leptin concentrations [23,24]. The higher leptin dose (500 μg/kg) significantly reduced serotonin catabolism, as indicated by decreased 5-hydroxyindoleacetic acid (5-HIAA) levels in both the hypothalamus and hippocampus, consistent with increased serotonergic availability [19]. These findings support leptin as a potential therapeutic target. However, the study also highlighted that leptin’s modulation of central and peripheral serotonin, along with other hormones, could influence treatment efficacy and potentially produce unwanted effects, particularly in comorbid conditions. This complexity underscores the need for careful consideration when developing leptin-based therapies for obesity, anxiety, and cognitive impairment [25,26,27,28].

Concurrently, plant-derived bioactive compounds have demonstrated promising anti-diabetic and anti-inflammatory activities, supporting their potential as nutraceutical interventions in metabolic disorders [29]. In this regard, natural products continue to be explored as viable alternatives for metabolic disorders, including diabetes and obesity [30]. Despite growing interest in nutraceutical interventions, the therapeutic potential of honey-derived compounds in obesity-related neurometabolic disorders remains underexplored. Natural food-derived products such as *Phoenix dactylifera* have demonstrated measurable pharmacological outcomes, including anti-inflammatory and analgesic effects in experimental models [31], lending support to the broader exploration of food-derived bioactive compounds such as honey glycoproteins as therapeutic agents. We previously observed that natural honey consumption caused weight loss in rats by altering the expression of lipid metabolism genes, such as *FABP1* and *LIPC*, and by increasing locomotor activity [32]. Honey glycoproteins and glycopeptides have previously been shown to exhibit immunomodulatory properties in vitro and to inhibit reactive oxygen species generation in zymogen-activated macrophages, indicating a potential tissue-protective function [33]. To substantiate this novelty claim, we conducted a systematic search of PubMed, Scopus, and Google Scholar using the terms “honey glycoproteins,” “honey proteins,” “MRJPs,” and “major royal jelly proteins,” combined with “obese rats,” “high-fat diet,” “leptin,” “serotonin,” and “anxiety.” This search returned no prior study that has isolated and administered honey glycoproteins/MRJPs in an HFD-induced obese rat model to simultaneously assess metabolic (leptin, TAG) and neurochemical (5-HT, 5-HIAA) outcomes, representing, to our knowledge, a previously unexplored area of investigation. This study, therefore, aims to assess the impact of natural honey and its glycoproteins on obesity and anxiety, and to elucidate the mechanisms by which these interventions ameliorate obesity-associated anxiety, with a focus on modulation of the leptin–serotonin axis.

## 2. Materials and Methods

### 2.1. Experimental Design

Before the start of this study, ethical approval was obtained in April 2018 from the Institutional Animal Care and Use Committee (IACUC) under Approved Study Protocol (ASP) # 2018-0002, and the study was carried out according to standard guidelines for animal care. Sixty male Wistar rats, weighing 120–140 g, were obtained from the animal house facility of the Dr. Panjwani Center for Molecular Medicine and Drug Research (PCMD), International Center for Chemical and Biological Sciences (ICCBS), University of Karachi. Each rat was housed individually to allow accurate measurement of individual food intake and was kept under controlled temperature (22 ± 2 °C), relative humidity (50–60%), and a 12:12 h light/dark cycle. To minimize potential isolation-induced stress, rats were provided with environmental enrichment (nesting material) and handled daily during the acclimatization period. The rats were acclimatized for one week, during which they had free access to a normal diet (ND) and plain drinking water. Animals were housed and handled according to the guidelines of the ‘Guide for the Care and Use of Laboratory Animals’ (The National Academies Press, Washington, DC, USA).

### 2.2. Composition of Diets

The ND contained 10% kcal fat, 70% kcal carbohydrate, and 20% kcal protein (Product #D12450, Research Diets Inc., New Brunswick, NJ, USA), whereas the HFD (Product #D12492, Research Diets Inc., New Brunswick, NJ, USA) contained 60% kcal fat, 20% kcal carbohydrate, and 20% kcal protein.

### 2.3. Dosage of the Honey and Honey Glycoproteins

Raw acacia honey, obtained from a local market in Karachi, was administered orally at two doses: a low dose (LHD) of 1 g/kg and a high dose (HHD) of 2 g/kg, as previously described [34]. Animals in the control groups received an oral dose of saline solution (0.9% NaCl). For the honey glycoproteins, protein precipitation was carried out using the salting-out method, as previously described [33]. The protein solution was administered intraperitoneally at two doses: a low protein dose (LPD) of 0.25 mg/kg and a high protein dose (HPD) of 0.5 mg/kg. These doses were calculated based on the protein yield (~0.125% of total honey solids), as previously reported [35].

### 2.4. Behavioral Activities

To assess locomotion and anxiety-like behavior, two behavioral tests were conducted: the elevated plus maze test and the open-field test. These tests were performed in a controlled environment—a soundproof, ventilated room maintained at a consistent temperature (26 ± 1 °C). To minimize stress or anxiety caused by relocation, animals were acclimatized to the test room for 1 h before testing began. All tests were conducted during the light phase, between 9 AM and 12 PM. Animals were tested in random order, and the observer remained blinded to the treatment groups until scoring was complete.

### 2.5. Elevated Plus Maze (EPM) Test

The EPM apparatus consists of a plus-shaped platform with four arms (two open and two closed). Each arm measures 12 cm in width and 50 cm in length. The arms are joined by a central square area measuring 12 cm × 12 cm and are elevated 60 cm above the floor. The two open arms have no boundary walls, whereas the closed arms have boundary walls 12 cm in height. This apparatus is used to assess anxiety-like behavior in animals. The number of entries into the open arms and the total duration spent there (in seconds) were recorded over 5 min. The duration spent in the open arms reflects anti-anxiety-like behavior. The EPM test was performed as previously described [36,37].

### 2.6. Open-Field Test

The open-field apparatus consists of a wall-enclosed square box (76 × 76 cm) with 42 cm high walls. The floor of the apparatus is divided into 25 equal squares. The open-field test assesses locomotor activity and anxiety-related behavior in rats. Each rat was placed in the central square of the box, and its movements were recorded for 5 min. The number of squares crossed with all four paws was recorded as a measure of locomotor activity. The box was cleaned with 70% ethanol after each animal was tested [37,38].

### 2.7. 5-HT and 5-HIAA Quantification

For 5-HT and 5-HIAA quantification, whole brain tissue was homogenized in 0.1 M perchloric acid (Merck, Darmstadt, Germany) (containing an internal standard) using a mechanical homogenizer, then centrifuged at 12,000 rpm for 20 min at 4 °C. The supernatant was collected and re-centrifuged at 12,000 rpm for a further 5 min. Quantification of 5-HT and 5-HIAA was performed by high-performance liquid chromatography (HPLC) using a Waters e2695 Separations Module coupled with a Waters 2465 electrochemical detector (Waters Corporation, Milford, MA, USA) operating at +0.8 V. Separation was achieved on an octadecylsilane (ODS, C18) reversed-phase column (150 mm × 4.6 mm, 5 μm particle size). The mobile phase consisted of 0.1 M sodium phosphate (Merck, Darmstadt, Germany) buffer (pH 3.0) containing 0.1 mM EDTA (Merck, Darmstadt, Germany), 1.2 mM sodium octyl sulfate (Merck, Darmstadt, Germany), and 10% methanol (Sigma Aldrich, St. Louis, MO, USA), delivered at a flow rate of 1.0 mL/min. The operating pressure was maintained at 2000–3000 psi. Standard solutions of 100 ng/mL were used for 5-HT and 5-HIAA, and in-house controls were used for quantification.

### 2.8. Blood Collection for Leptin and TAG Test

Immediately following decapitation, blood specimens were collected between 09:00 and 11:00 h into sterilized glass tubes. Rats were not fasted before blood collection. For serum separation, blood was allowed to clot at room temperature for 30 min, then centrifuged at 3000 rpm for 30 min. The upper serum layer was carefully collected and stored at −80 °C until analysis. All assays were performed in duplicate.

#### 2.8.1. Quantification of Serum Leptin

Serum leptin concentration was measured using a commercially available ELISA kit (Invitrogen Inc., Carlsbad, CA, USA), according to the manufacturer’s instructions.

#### 2.8.2. TAG Quantification

Serum triacylglycerol (TAG) was quantified using a commercially available kit (SPINREACT, Girona, Spain), according to the manufacturer’s instructions.

### 2.9. Statistical Analysis

Statistical analyses of neurochemical, biochemical, and behavioral data were performed using two-way or three-way analysis of variance (ANOVA), as appropriate. Results are presented as mean ± standard deviation (SD). Tukey’s post hoc test was used for individual comparisons. Statistical analyses were performed using IBM SPSS software, version 23.0.

### 2.10. Grouping of Animals

After acclimatization, 60 rats were divided into two major groups: 30 rats on the ND and 30 rats on the HFD for four weeks to produce diet-induced obesity. Each of the ND and HFD groups was then further divided into five sub-groups (*n* = 6 each), as shown below, constituting a total of 10 groups (Table 1; Supplementary Figure S1). The number of rats per group was determined using the “resource equation” method to justify statistical power, as previously described [39].

The HFD was started on day 1 of week 1 and continued for 4 weeks. Honey or honey protein supplementation, along with the HFD or ND, was started on day 1 of week 5 and continued for the next 4 weeks. Saline was given to the control groups along with their respective ND or HFD. Food intake was recorded daily, and body weight was measured weekly from week 1 to week 8. The open-field and elevated plus maze (EPM) tests were also performed weekly throughout the study (weeks 1–8). Animals were sacrificed by decapitation upon completion of the behavioral tests at the end of week 8. After sacrifice, blood and whole brain tissue were collected for further analysis, as described in Supplementary Figure S2.

## 3. Results

The results for the different parameters are presented in three tiers: the impact of diet (ND vs. HFD), the impact of natural honey, and the impact of honey glycoproteins on these parameters.

### 3.1. Impact of HFD on Food Intake and Body Weight Changes

**Figure 1:**
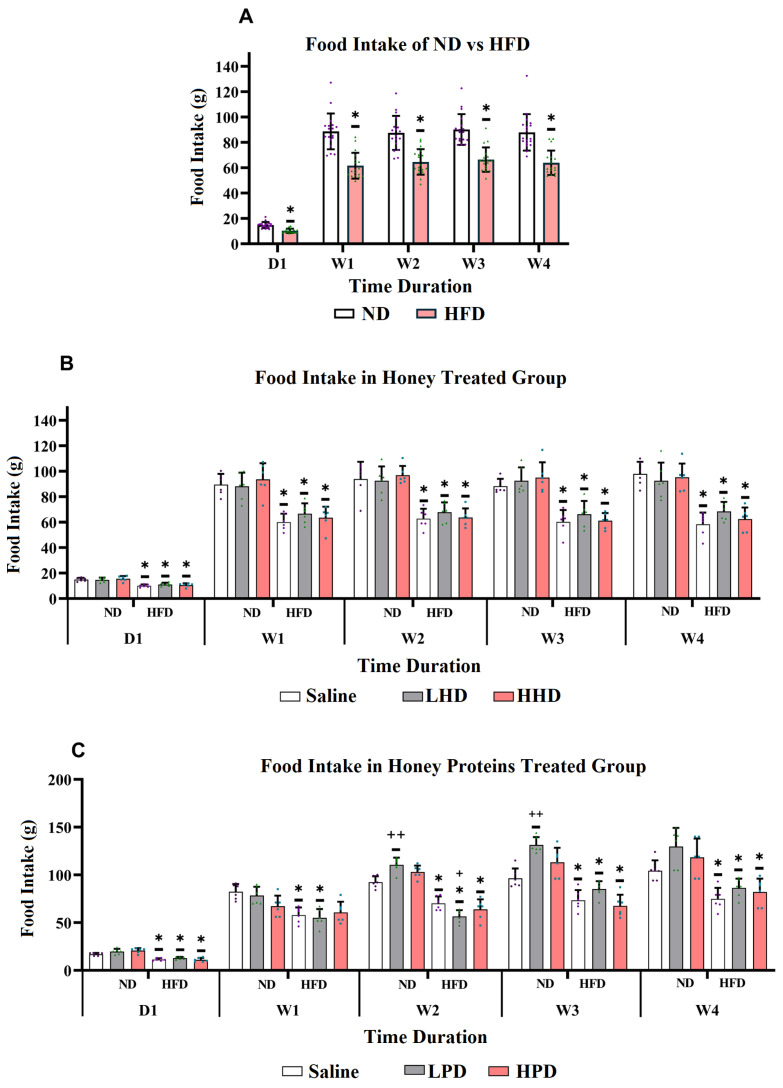
(**A**–**C**): Food intake (g) in the experimental rats. (**A**) Food intake by rats on ND (*n* = 30) or HFD (*n* = 30). Values are means ± SD. Significant differences by Tukey’s test: * *p* < 0.01 from the respective ND, following two-way ANOVA (repeated-measures design). (**B**) Food intake of ND and HFD rats treated with honey (HHD (*n* = 6) and LHD (*n* = 6)). (**C**) Food intake of ND and HFD rats treated with honey proteins (HPD (*n* = 6) and LPD (*n* = 6)). Significant differences by Tukey’s test: * *p* < 0.01 from the respective ND, + *p* < 0.05, ++ *p* < 0.01 from HFD saline animals, following three-way ANOVA (repeated-measures design). (D = Day, W = Week).

Food intake was monitored daily, and body weight was monitored weekly for 8 weeks. Figure 1A shows the quantity of food consumed (in grams) by the two groups of rats given free access to the ND or HFD for four weeks. The data, analyzed by two-way ANOVA (repeated-measures design), showed a significant effect of HFD on food intake (F = 56.1, df = 1, 34, *p* < 0.01). The effects of repeated measures (weekly monitoring) (F = 509.7, df = 4, 136, *p* < 0.01) and the interaction between repeated measures and HFD (F = 12.7, df = 4, 136, *p* < 0.01) were also significant. Tukey’s post hoc test revealed that cumulative weekly food intake was lower in the HFD animals than in the ND animals from week 1 to week 4 (*p* < 0.01).

Figure 1B shows the effects of the two honey doses on food intake. Following a three-way ANOVA (repeated-measures design), the data revealed significant effects of repeated measure (weekly monitoring) (F = 559.8, df = 4, 120, *p* < 0.01), as well as a significant interaction between repeated measure × HFD (F = 21.0, df = 4, 120, *p* < 0.01). HFD had a substantial effect (F = 182.5, df = 1, 30, *p* < 0.01). There were no significant effects of honey treatment (F = 0.7, df = 2, 30, *p* > 0.05) or HFD × honey interaction (F = 1.5, df = 2, 30, *p* > 0.05). The interactions between repeated measure × HFD × honey treatment (F = 0.5, df = 8, 120, *p* > 0.05) and repeated measure × honey treatment (F = 0.24, df = 8, 120, *p* > 0.05) were also not significant. Post hoc analysis showed no significant effects of HHD or LHD on food intake in HFD-treated animals compared with saline-treated animals. Similarly, HHD and LHD had non-significant effects (*p* > 0.05) on food intake in ND-treated animals compared with saline-treated animals.

Figure 1C shows the effects of honey proteins on food intake. The data, analyzed by three-way ANOVA (repeated-measures design), showed significant effects of repeated measure (weekly monitoring) (F = 452.6, df = 4, 120, *p* < 0.01) and a significant interaction between repeated measure × HFD (F = 19.8, df = 4, 120, *p* < 0.01). The effects of HFD (F = 307.5, df = 1, 30, *p* < 0.01), honey protein treatment (F = 10.4, df = 2, 30, *p* < 0.01), and the HFD × honey protein interaction (F = 6.7, df = 2, 30, *p* < 0.01) were also significant. The interactions between repeated measure × honey protein treatment (F = 5.1, df = 8, 120, *p* < 0.01) and repeated measure × HFD × honey protein treatment (F = 2.5, df = 8, 120, *p* < 0.01) were also significant. Post hoc analysis showed that food intake was significantly lower in the HFD rats than in the ND rats. The LPD and HPD treatments had no significant impact on food intake within each group weekly, except in weeks 2 and 3. In week 2, LPD significantly reduced food intake in the HFD group, while in weeks 2 and 3, LPD significantly increased food intake in the ND rats.

**Figure 2:**
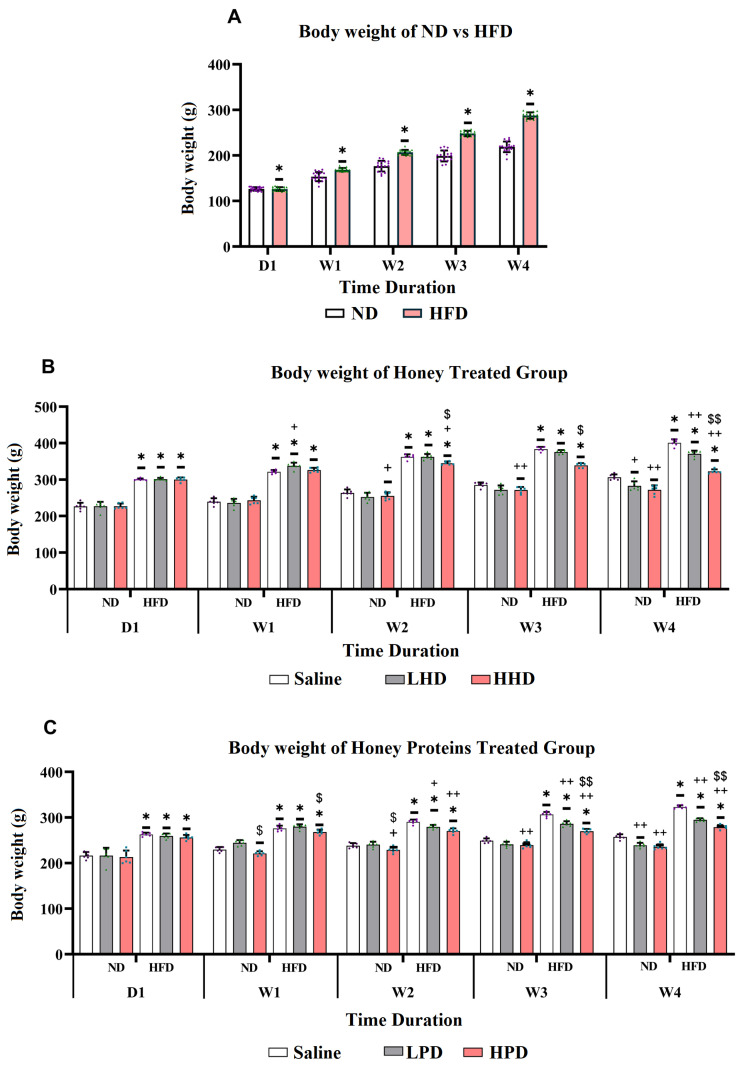
(**A**–**C**): Body weights of the experimental rats during the course of the study. (**A**) Body weight of ND (*n* = 30) and HFD (*n* = 30) rats. Values are means ± SD. Significant differences by Tukey’s test: * *p* < 0.01 from respective ND animals, following two-way ANOVA (repeated-measures design). (**B**) Body weight of ND and HFD rats treated with honey (HHD (*n* = 6) and LHD (*n* = 6)). Values are means ± SD. Significant differences by Tukey’s test: * *p* < 0.01 from respective ND animals, + *p* < 0.05, ++ *p* < 0.01 from HFD saline animals, $ *p* < 0.05 from respective HHD animals, following three-way ANOVA (repeated-measures design). (**C**) Body weight of ND and HFD rats treated with honey proteins (HPD (*n* = 6) and LPD (*n* = 6)). Values are means ± SD. Significant differences by Tukey’s test: * *p* < 0.05 from respective ND animals, + *p* < 0.05, ++ *p* < 0.01 from HFD saline animals, $ *p* < 0.05, $$ *p* < 0.01 from respective LPD animals, following three-way ANOVA (repeated-measures design). (D = Day, W = Week).

Figure 2A shows weekly changes in body weight in the two groups of rats given free access to either the ND or HFD for four weeks. HFD (F = 217.5, df = 1, 34, *p* < 0.01) and repeated measures (weekly monitoring) (F = 2991.8, df = 4, 136, *p* < 0.01) had significant effects, as determined by two-way ANOVA (repeated-measures design). Weekly monitoring and HFD also showed a significant interaction (F = 222.9, df = 4, 136, *p* < 0.01). Post hoc tests showed that body weights of the ND and HFD rats were similar on day 1 of the experiment, but weekly weight gain was greater in the HFD than in the ND animals from week 1 (*p* < 0.01) to week 4 (*p* < 0.01).

Figure 2B shows the effects of the two honey doses on body weight changes. The data, analyzed by three-way ANOVA (repeated-measures design), showed significant effects of HFD (F = 1252.9, df = 1, 30, *p* < 0.01), repeated measure (weekly monitoring) (F = 922.4, df = 4, 120, *p* < 0.01), and a significant interaction between repeated measure × HFD (F = 35.3, df = 4, 120, *p* < 0.01). The effects of honey treatment (F = 20.5, df = 2, 30, *p* < 0.01) and the HFD × honey treatment interaction (F = 7.7, df = 2, 30, *p* < 0.01) were also significant. The interactions between repeated measure × honey treatment (F = 69.1, df = 8, 120, *p* < 0.01) and repeated measure × HFD × honey treatment (F = 13.5, df = 8, 120, *p* < 0.01) were also significant. Post hoc analysis by Tukey’s test showed that HHD decreased body weight from week 3, and LHD from week 4, in ND animals (*p* < 0.01) compared with the saline group. Similarly, HHD significantly decreased body weight in HFD animals from week 2 (*p* < 0.01) compared with saline treatment. LHD initially increased body weight in week 1 in the HFD group, then decreased it from week 4 onward. HHD produced a significantly greater decrease in body weight than LHD in HFD animals.

Figure 2C shows the effects of honey proteins on body weight changes. The data, analyzed by three-way ANOVA (repeated-measures design), showed significant effects of HFD (F = 1339.2, df = 1, 30, *p* < 0.01), repeated measure (weekly monitoring) (F = 288.2, df = 4, 120, *p* < 0.01), and a significant interaction between repeated measure × HFD (F = 10.4, df = 4, 120, *p* < 0.01). The effects of honey protein treatment (F = 66.5, df = 2, 30, *p* < 0.01) and the HFD × honey protein interaction (F = 9.0, df = 2, 30, *p* < 0.01) were also significant. The interactions between repeated measure × honey protein treatment (F = 27.6, df = 8, 120, *p* < 0.01) and repeated measure × HFD × honey protein treatment (F = 8.4, df = 8, 120, *p* < 0.01) were also significant. Post hoc analysis showed that LPD and HPD decreased body weight in both ND and HFD animals compared with the respective saline groups. HPD produced a greater reduction in body weight than LPD in the HFD group from week 1. Similarly, HPD decreased body weight in weeks 1 and 2 in the ND animals. LPD initially increased body weight in week 1, then decreased it from week 4 (*p* < 0.01) in the ND group and from week 2 in the HFD group (*p* < 0.05 in week 2, *p* < 0.01 from week 3 onward).

Notably, the HFD rats consumed less food than the ND rats; however, they showed significantly greater weight gain (*p* < 0.01). Caloric intake was calculated from the diet and treatment consumed, and was comparable (*p* > 0.05) between the two groups (Supplementary Figure S3).

### 3.2. Impact of HFD, Honey, and Its Glycoproteins on the Elevated Plus Maze (EPM) Test

In the EPM test, the number of entries into and time spent in the open arms were recorded and analyzed using two-way or three-way ANOVA, as appropriate.

**Figure 3:**
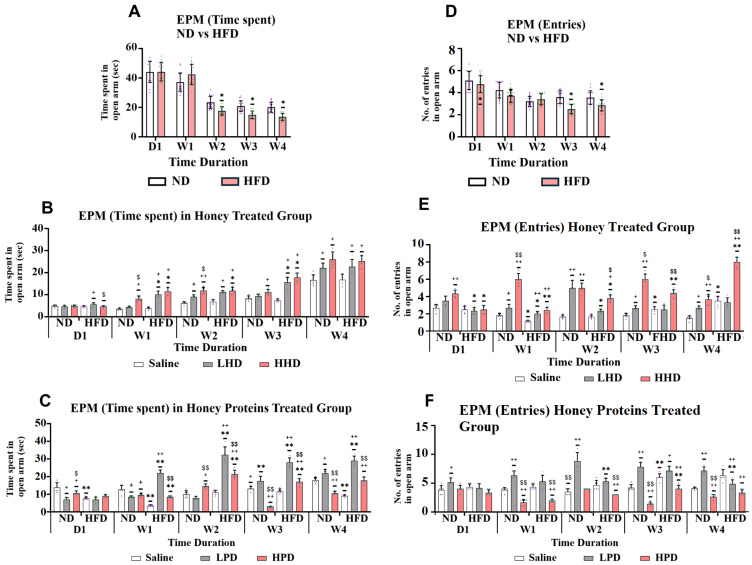
(**A**–**F**): Time spent in (**A**–**C**) and number of entries into (**D**–**F**) the open arms of the elevated plus maze. Values are means ± SD. (**A**) Time spent by ND (*n* = 30) and HFD (*n* = 30) rats in the open arms. Significant differences by Tukey’s test: * *p* < 0.05, ** *p* < 0.01 from respective ND animals, following two-way ANOVA (repeated-measures design). (**B**) Time spent in the open arms by ND and HFD rats treated with honey (LHD (*n* = 6) and HHD (*n* = 6)). (**C**) Time spent in the open arms by ND and HFD rats treated with honey proteins (HPD (*n* = 6) and LPD (*n* = 6)). (**D**) Number of entries by ND (*n* = 30) and HFD (*n* = 30) rats into the open arms. (**E**) Number of entries into the open arms by ND and HFD rats treated with honey (LHD (*n* = 6) and HHD (*n* = 6)). Values are means ± SD. Significant differences by Tukey’s test: * *p* < 0.05, ** *p* < 0.01 from respective ND animals, + *p* < 0.05, ++ *p* < 0.01 from HFD saline animals, $ *p* < 0.05, $$ *p* < 0.01 from respective HHD animals, following three-way ANOVA (repeated-measures design). (**F**) Number of entries into the open arms by ND and HFD rats treated with honey proteins (LPD (*n* = 6) and HPD (*n* = 6)). Values are means ± SD. Significant differences by Tukey’s test: * *p* < 0.05, ** *p* < 0.01 from respective ND animals, + *p* < 0.05, ++ *p* < 0.01 from HFD saline animals, $ *p* < 0.05, $$ *p* < 0.01 from respective HPD animals, following three-way ANOVA (repeated-measures design).

Figure 3A shows the effect of HFD on anxiety-like behavior (time spent in the open arms) in rats given free access to either ND or HFD for four weeks. HFD (F = 9.1, df = 1, 34, *p* < 0.01) and repeated measures (weekly monitoring) (F = 283.9, df = 4, 136, *p* < 0.01) had significant effects, as determined by two-way ANOVA (repeated-measures design). The interaction between weekly monitoring and HFD was also significant (F = 10.9, df = 4, 136, *p* < 0.01). Post hoc tests showed that EPM activity in the ND and HFD rats was comparable on day 1, but weekly decreases were greater in the HFD than in the ND rats from week 2 (*p* < 0.05) to week 4 (*p* < 0.01).

Figure 3B shows the effects of the two honey doses on time spent in the open arms. The data, analyzed by three-way ANOVA (repeated-measures design), revealed significant effects of repeated measure (weeks) (F = 662.4, df = 4, 120, *p* < 0.01) and a significant interaction between repeated measure × diet (F = 12.8, df = 4, 120, *p* < 0.01). Both honey treatment (F = 202.3, df = 2, 30, *p* < 0.01) and diet (F = 53.5, df = 1, 30, *p* < 0.01) had significant effects, as did the diet × honey interaction (F = 19.7, df = 2, 30, *p* < 0.01). The interactions between repeated measure × diet × honey (F = 4.8, df = 8, 120, *p* < 0.01) and repeated measure × honey (F = 14.5, df = 8, 120, *p* < 0.01) were also significant. Post hoc analysis showed that honey-treated rats spent significantly more time (*p* < 0.01) in the open arms compared with both the HFD and ND saline groups. HHD produced a greater increase in time spent in the open arms than LHD in weeks 1 and 2 in the ND animals. In the HFD group, both HHD and LHD significantly increased time spent in the open arms (*p* < 0.05 and *p* < 0.01, respectively). These data indicate that honey treatment decreased anxiety-like behavior in both HFD and ND animals.

Figure 3C shows the effects of honey proteins on time spent in the open arms. The data, analyzed by three-way ANOVA (repeated-measures design), showed significant effects of repeated measure (weeks) (F = 150.2, df = 4, 120, *p* < 0.01) and a significant interaction between repeated measure × diet (F = 82.5, df = 4, 120, *p* < 0.01). The effects of honey proteins (F = 231.2, df = 2, 30, *p* < 0.01) and diet (F = 169.5, df = 1, 30, *p* < 0.01) were also significant, as was the diet × honey proteins interaction (F = 269.2, df = 2, 30, *p* < 0.01). The interactions between repeated measure × honey proteins (F = 60.4, df = 8, 120, *p* < 0.01) and repeated measure × diet × honey proteins (F = 27.6, df = 8, 120, *p* < 0.01) were also significant. Post hoc analysis showed that honey protein-treated rats spent significantly more time (*p* < 0.01) in the open arms compared with both the HFD and ND saline groups. LPD produced a greater increase in time spent in the open arms than HPD in weeks 1–4 in the HFD animals, whereas in the ND animals, LPD showed significant effects in weeks 3 and 4 compared with HPD. These data indicate that honey protein treatment decreased anxiety-like behavior in both HFD and ND animals.

Figure 3D shows the effect of HFD on the number of entries into the open arms in rats given free access to ND or HFD for four weeks. HFD (F = 30.8, df = 1, 34, *p* < 0.01) and repeated measures (weekly monitoring) (F = 58.8, df = 4, 136, *p* < 0.01) had significant effects on the number of entries, as determined by two-way ANOVA (repeated-measures design). The interaction between weekly monitoring and HFD was also significant (F = 5.31, df = 4, 136, *p* < 0.01). Post hoc tests showed that the number of entries by the ND and HFD rats was comparable from day 1 to week 2, but in weeks 3 and 4, the HFD rats showed significantly decreased activity (*p* < 0.01) compared with the ND rats.

Figure 3E shows the effect of the honey doses on the number of entries into the open arms. The data, analyzed by three-way ANOVA (repeated-measures design), revealed significant effects of repeated measure (weeks) (F = 31.1, df = 4, 120, *p* < 0.01) and a significant interaction between repeated measure × diet (F = 119.9, df = 4, 120, *p* < 0.01). Both honey treatment (F = 537.6, df = 2.30, *p* < 0.01) and diet (F = 39.7, df = 1, 30, *p* < 0.01) had significant effects, as did the diet × honey interaction (F = 36.3, df = 2, 30, *p* < 0.01). The interactions between repeated measure × diet × honey (F = 30.9, df = 8, 120, *p* < 0.01) and repeated measure × honey (F = 26.8, df = 8, 120, *p* < 0.01) were also significant. Post hoc analysis showed that the HFD animals entered the open arms significantly less often (*p* < 0.05) than the ND animals in week 1, but significantly more often by week 4. In both ND and HFD rats, HHD produced significantly more entries into the open arms (*p* < 0.01) than LHD (Figure 3E). These findings suggest that HHD decreases anxiety-like behavior in both ND and HFD rats.

Figure 3F shows the effect of the honey protein doses on the number of entries into the open arms. The data, analyzed by three-way ANOVA (repeated-measures design), showed significant effects of repeated measure (weeks) (F = 25.7, df = 4, 120, *p* < 0.01) and a significant interaction between repeated measure × diet (F = 18.0, df = 4, 120, *p* < 0.01). The effect of honey proteins (F = 211.9, df = 2, 30, *p* < 0.01) was significant, while the effect of diet (F = 0.06, df = 1, 30, *p* > 0.05) was not. The diet × honey protein interaction was significant (F = 44.4, df = 2, 30, *p* < 0.01), as were the interactions between repeated measure × honey proteins (F = 22.2, df = 8, 120, *p* < 0.01) and repeated measure × diet × honey proteins (F = 7.3, df = 8, 120, *p* < 0.01). Post hoc analysis showed significantly more entries into the open arms (*p* < 0.05) from week 1 to week 4 in the ND rats. The number of entries in the HFD animals was significantly increased (*p* < 0.01) in weeks 3 and 4 compared with the ND animals, while more entries were observed in LPD-treated HFD rats in weeks 1–3. LPD produced a significantly greater increase (*p* < 0.01) in entries than HPD in both the HFD and ND animals (Figure 3F). These data indicate that LPD improved anxiety-like behavior in both ND and HFD rats.

Honey treatment increased time spent and entries in the open arms (*p* < 0.01), indicating an anxiolytic effect, with HHD producing a greater anxiolytic effect than LHD in ND rats from week 1 to week 2. Honey proteins (LPD) showed a superior anxiolytic effect in HFD rats (*p* < 0.01) (Figure 3).

### 3.3. Impact of Honey and Its Glycoproteins on the Open-Field Test

**Figure 4:**
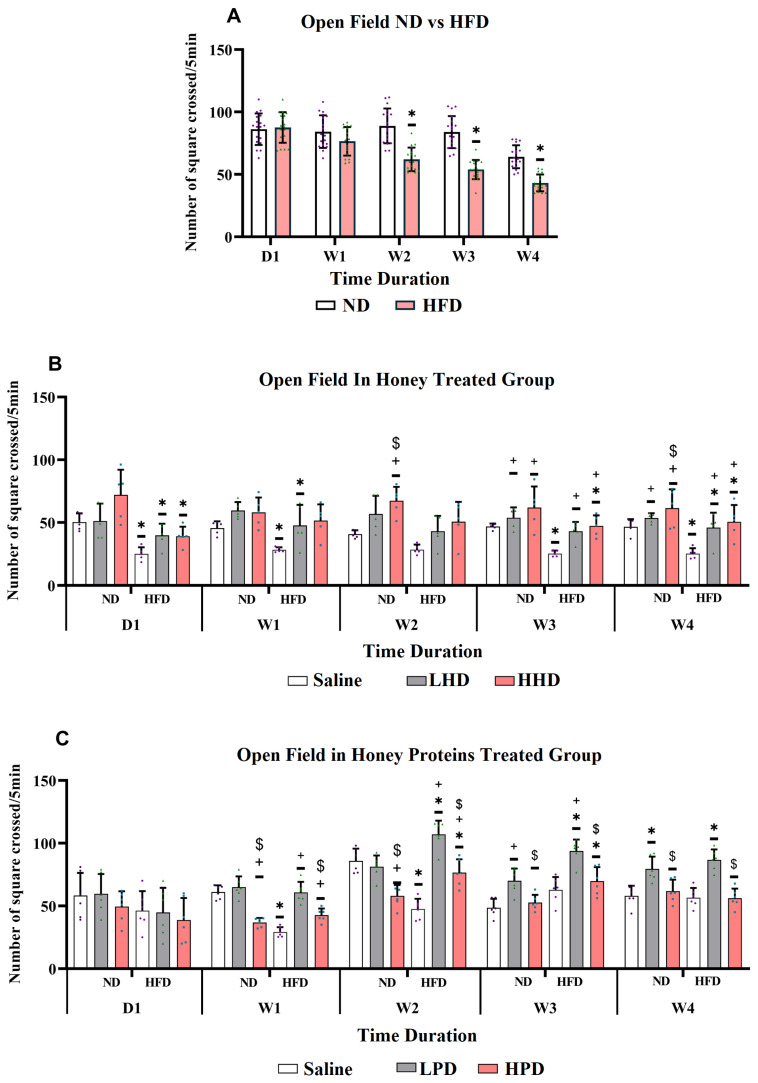
(**A**–**C**): Open-field activity of ND and HFD rats treated with honey (HHD and LHD) and honey proteins (LPD and HPD). Values are means ± SD. (**A**) Open-field activity of ND (*n* = 30) and HFD (*n* = 30) rats. Significant differences by Tukey’s test: * *p* < 0.01 from respective ND animals, following two-way ANOVA (repeated-measures design). (**B**) Open-field activity of ND and HFD rats treated with honey (LHD (*n* = 6) and HHD (*n* = 6)). Significant differences by Tukey’s test: * *p* < 0.05 from respective ND animals, + *p* < 0.05 from HFD saline animals, $ *p* < 0.05 from respective HHD animals, following three-way ANOVA (repeated-measures design). (**C**) Open-field activity of ND and HFD rats treated with honey proteins (LPD (*n* = 6) and HPD (*n* = 6)). Significant differences by Tukey’s test: * *p* < 0.05 from respective ND animals, + *p* < 0.05 from HFD saline animals, $ *p* < 0.05 from respective HPD animals, following three-way ANOVA (repeated-measures design).

In the open-field test, the number of squares crossed by a rat is recorded to assess its locomotor activity in a novel environment. Locomotor activity was reduced in the HFD rats from week 2 onward (*p* < 0.01). Both honey doses improved activity in HFD rats (*p* < 0.01), with HHD showing greater effects in ND rats. LPD treatment enhanced activity for a longer duration (weeks 1–4) than HPD (weeks 1–3) (Figure 4).

Figure 4A shows the effects of HFD on open-field activity (number of squares crossed in a novel environment). HFD (F = 68.07, df = 1, 34, *p* < 0.01) and repeated measures (weekly monitoring) (F = 53.2, df = 4, 136, *p* < 0.01) had significant effects, as determined by two-way ANOVA (repeated-measures design). The interaction between weekly monitoring and HFD was also significant (F = 14.5, df = 4, 136, *p* < 0.01). Post hoc tests showed that exploratory activity in the ND and HFD rats was comparable on day 1, but weekly declines were greater in the HFD than in the ND rats from week 2 (*p* < 0.01) through week 4 (*p* < 0.01).

Figure 4B shows the effects of the two honey doses on open-field activity in the ND and HFD rats. The data, analyzed by three-way ANOVA (repeated-measures design), showed significant effects of repeated measure (weeks) (F = 53.5, df = 4, 120, *p* < 0.01), diet (F = 165.5, df = 1, 30, *p* < 0.01), and honey (F = 87.4, df = 2, 30, *p* < 0.01). The diet × honey interaction was also significant (F = 3.5, df = 2, 30, *p* < 0.05). The interactions between repeated measure × diet (F = 2.1, df = 4, 120, *p* > 0.05) and repeated measure × diet × honey (F = 1.9, df = 8, 120, *p* > 0.05) were not significant. The repeated measure × honey interaction was significant (F = 18.7, df = 8, 120, *p* < 0.01). Post hoc analysis showed that both HHD and LHD increased locomotor activity in HFD-treated animals from week 1 to week 4 (*p* < 0.01). HHD produced a significantly greater increase in locomotor activity than LHD in ND-treated animals on weeks 2 and 4 (*p* < 0.01).

Figure 4C shows the effects of the honey protein doses on open-field activity. The data, analyzed by three-way ANOVA (repeated-measures design), showed significant effects of repeated measures (weeks) (F = 50.8, df = 4, 120, *p* < 0.01) and honey proteins (F = 47.4, df = 2, 30, *p* < 0.01). The effect of diet (F = 0.05, df = 1, 30, *p* > 0.05) was not significant. The diet × honey protein interaction was significant (F = 12.1, df = 2, 30, *p* < 0.01), as were the interactions between repeated measure × diet (F = 13.8, df = 4, 120, *p* < 0.01), repeated measure × honey proteins (F = 4.4, df = 8, 120, *p* < 0.01), and repeated measure × diet × honey proteins (F = 7.7, df = 8, 120, *p* < 0.01). Post hoc analysis showed that HFD significantly reduced locomotor activity compared with ND rats in weeks 1 and 2. LPD treatment significantly increased activity in the HFD rats from week 1 to week 4 (*p* < 0.01) and in the ND rats from week 3 to week 4 (*p* < 0.01). HPD significantly increased locomotor activity in both the ND and HFD rats from week 1 to week 3.

### 3.4. Impact of Honey and Its Glycoproteins on Serum TAG Levels

**Figure 5:**
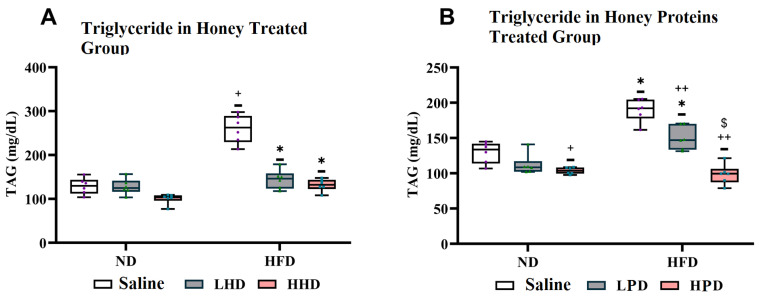
(**A**,**B**): Serum triacylglycerol (TAG) concentrations in ND and HFD rats treated with honey (HHD and LHD) and honey proteins (LPD and HPD). (**A**) Serum TAG concentrations in ND and HFD rats treated with honey (HHD (*n* = 6) and LHD (*n* = 6)). Significant differences by Tukey’s test: * *p* < 0.05 from respective ND animals, + *p* < 0.01 from respective saline animals, following two-way ANOVA. (**B**) Serum TAG concentrations in ND and HFD rats treated with honey proteins (HPD (*n* = 6) and LPD (*n* = 6)). Values are means ± SD. Significant differences by Tukey’s test: * *p* < 0.05 from respective ND animals, ++ *p* < 0.01 from respective saline animals, $ *p* < 0.01 from respective LPD animals, following two-way ANOVA.

There was a significant elevation in serum TAG levels (*p* < 0.01) in the HFD rats compared with the ND rats. Both honey and its glycoproteins significantly reduced TAG in the HFD rats (*p* < 0.01) (Figure 5). The efficacy of both interventions was greater in the HFD than in the ND groups (*p* < 0.01).

Figure 5A shows the effects of honey treatment for four weeks on serum TAG levels. The data, analyzed by two-way ANOVA, showed significant effects of HFD (F = 76.3, df = 1, 30, *p* < 0.01), honey treatment (F = 47.6, df = 2, 30, *p* < 0.01), and the HFD × honey treatment interaction (F = 27.8, df = 2, 30, *p* < 0.01). Post hoc analysis by Tukey’s test indicated higher serum TAG levels in the HFD than in the ND rats. Both high and low doses of honey decreased serum TAG levels in the HFD-treated rats, whereas the effects of high and low honey doses in the ND-treated rats were not significant.

Figure 5B shows the effects of honey protein treatment for four weeks on serum TAG levels in the ND and HFD rats. The data, analyzed by two-way ANOVA, showed significant effects of HFD (F = 43.1, df = 1, 30, *p* < 0.01), honey protein treatment (F = 51.0, df = 2, 30, *p* < 0.01), and the HFD × honey protein treatment interaction (F = 16.6, df = 2, 30, *p* < 0.01). Post hoc analysis indicated higher serum TAG levels in the HFD rats compared with the ND rats. TAG levels decreased in both the HFD and ND rats following honey protein treatment, with a greater effect observed in the HFD rats than in the ND rats.

### 3.5. Impact of Honey and Its Glycoproteins on Serum Leptin Levels

There was a significant increase in serum leptin levels (*p* < 0.01) in the HFD rats compared with the ND rats. Honey (HHD) and its glycoproteins (HPD) significantly reduced leptin levels in rats on both diets (*p* < 0.01), with greater reductions observed in the HFD rats (*p* < 0.01 for interactions).

**Figure 6:**
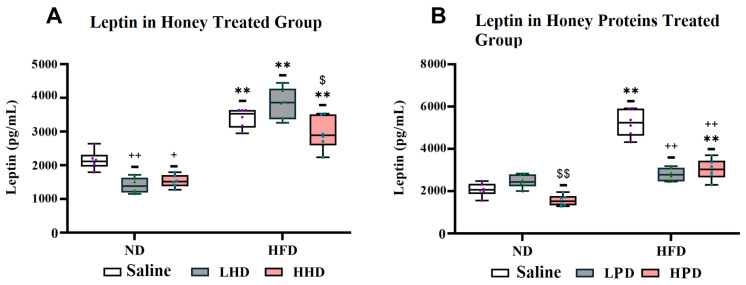
(**A**,**B**): Serum leptin concentrations in ND and HFD rats treated with honey (HHD and LHD) and honey proteins (LPD and HPD). (**A**) Serum leptin concentrations in ND and HFD rats treated with honey (HHD and LHD). Significant differences by Tukey’s test: ** *p* < 0.01 from respective ND animals, + *p* < 0.05, ++ *p* < 0.01 from respective saline animals, $ *p* < 0.01 from respective LHD animals, following two-way ANOVA. (**B**) Serum leptin concentrations in ND and HFD rats treated with honey proteins (HPD and LPD). Significant differences by Tukey’s test: ** *p* < 0.01 from respective ND animals, ++ *p* < 0.01 from respective saline animals, $$ *p* < 0.01 from respective LPD animals, following two-way ANOVA.

Figure 6A shows changes in serum leptin levels in the ND, HFD, and honey-treated animals. Two-way ANOVA revealed a significant effect of HFD (F = 223.3, df = 1, 30, *p* < 0.01) and a significant effect of honey treatment (F = 7.6, df = 2, 30, *p* < 0.01). The interaction between diet and honey treatment was also significant (F = 10.2, df = 2, 30, *p* < 0.01). Post hoc analysis showed that HHD reduced serum leptin levels in both the ND and HFD rats compared with the LHD and saline groups.

Figure 6B shows changes in serum leptin levels in the ND and HFD animals treated with honey proteins. Two-way ANOVA revealed significant effects of HFD (F = 145.6, df = 1, 30, *p* < 0.01) and honey protein treatment (F = 35.6, df = 2, 30, *p* < 0.01), as well as a significant HFD × honey protein treatment interaction (F = 36.1, df = 2, 30, *p* < 0.01). Tukey’s post hoc test showed significantly higher leptin concentrations in the HFD rats than in the ND rats. Both doses of honey proteins significantly decreased leptin levels in the HFD-treated rats. HPD also significantly decreased leptin levels in the ND animals compared with the ND saline animals.

### 3.6. Impact of Honey and Its Glycoproteins on Serotonin Metabolism

**Figure 7:**
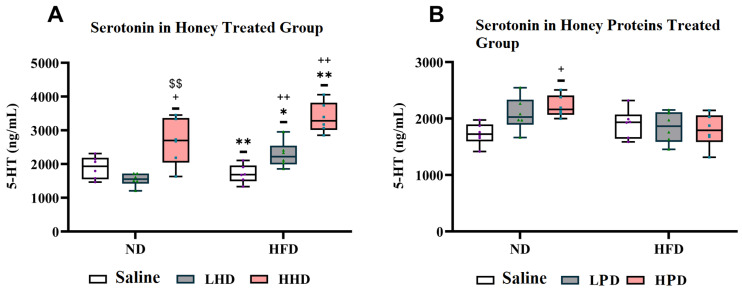
(**A**,**B**): Concentration of 5-HT in the whole brain of ND and HFD rats treated with honey (HHD and LHD) and honey proteins (LPD and HPD). (**A**) 5-HT concentrations in ND and HFD rats treated with honey (HHD (*n* = 6) and LHD (*n* = 6)). Significant differences by Tukey’s test: * *p* < 0.05, ** *p* < 0.01 from respective ND animals, ++ *p* < 0.01 from HFD saline animals, $$ *p* < 0.01 from respective HHD animals, following two-way ANOVA. (**B**) 5-HT concentrations in ND and HFD rats treated with honey proteins (LPD (*n* = 6) and HPD (*n* = 6)), + *p* < 0.05. Significant differences by Tukey’s test: * *p* < 0.05 from respective ND saline animals, following two-way ANOVA.

Honey treatment (LHD and HHD) increased central 5-HT levels in the HFD rats (*p* < 0.01) and in the ND rats (HHD only) (*p* < 0.01). Honey proteins (HPD) elevated 5-HT only in the ND rats (*p* < 0.05), with a significant diet × treatment interaction (*p* < 0.05) (Figure 7).

Figure 7A shows the effect of honey treatment on whole-brain 5-HT levels in the HFD and ND rats. HFD had a significant effect on 5-HT concentrations, as shown by two-way ANOVA (F = 9.2, df = 1, 30, *p* < 0.01). Honey treatment also had a significant effect (F = 31.4, df = 2, 30, *p* < 0.01), as did the diet × honey treatment interaction (F = 4.7, df = 2, 30, *p* < 0.01). Post hoc analysis showed a trend toward lower 5-HT levels in the HFD-treated rats than in the ND-treated rats, which did not reach statistical significance. In the HFD group, both HHD and LHD significantly increased 5-HT levels, whereas in the ND group, only HHD produced a significant increase. HHD produced a significantly greater increase in 5-HT levels than LHD in the HFD rats, and a significantly greater increase than in the ND rats treated with HHD.

Figure 7B shows the effect of honey protein treatment on whole-brain 5-HT levels in the HFD and ND rats. The data, analyzed by two-way ANOVA, showed non-significant effects of diet (F = 3.6, df = 1, 30, *p* > 0.05) and honey protein treatment (F = 1.8, df = 2, 30, *p* > 0.05). The diet × honey protein treatment interaction was significant (F = 4.3, df = 2, 30, *p* < 0.05). Post hoc analysis showed that HPD significantly increased 5-HT levels in the ND rats.

### 3.7. Impact of Honey and Its Glycoproteins on 5-HIAA Levels

**Figure 8:**
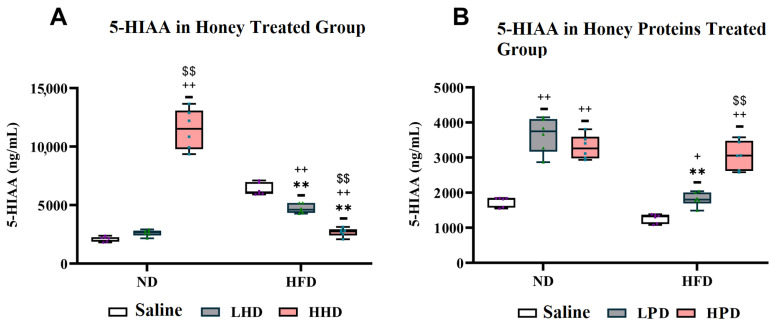
(**A**,**B**): Concentration of 5-HIAA (pg/mL) in the whole brain of ND and HFD rats treated with honey (HHD and LHD) and honey proteins (LPD and HPD). (**A**) 5-HIAA concentrations in ND and HFD rats treated with honey (HHD (*n* = 6) and LHD (*n* = 6)). Significant differences by Tukey’s test: ** *p* < 0.01 from respective ND animals, ++ *p* < 0.01 from HFD saline animals, $$ *p* < 0.01 from respective HHD animals, following two-way ANOVA. (**B**) 5-HIAA concentrations in ND and HFD rats treated with honey proteins (LPD (*n* = 6) and HPD (*n* = 6)). Significant differences by Tukey’s test: ** *p* < 0.01 from respective ND animals, + *p* < 0.05, ++ *p* < 0.01 from HFD saline animals, $$ *p* < 0.01 from respective HPD animals, following two-way ANOVA.

Honey treatment resulted in a significant increase in 5-HIAA levels, with HHD showing a more pronounced effect than LHD. With honey protein treatment, a greater increase in 5-HIAA was observed with HPD than with LPD (*p* < 0.01). Following both honey and honey protein treatments, the increase in 5-HIAA was greater in the HFD rats than in the ND rats, with significant diet × treatment interactions (*p* < 0.01) (Figure 8).

Figure 8A shows the effect of honey treatment on whole-brain 5-HIAA levels. The data, analyzed by two-way ANOVA, showed a non-significant effect of diet (F = 2.6, df = 1, 30, *p* > 0.05), but significant effects of honey treatment (F = 209.9, df = 2, 30, *p* < 0.01) and the diet × honey treatment interaction (F = 19.0, df = 2, 30, *p* < 0.01). Tukey’s post hoc test showed that both HHD and LHD significantly increased 5-HIAA levels in the HFD group, with HHD producing a significantly greater increase than LHD. HHD treatment also increased 5-HIAA levels in the ND rats, with a significantly greater increase observed in the ND group than in the HFD rats treated with HHD.

5-HIAA is the primary metabolite of 5-HT. Figure 8B shows the effect of honey protein treatment on whole-brain 5-HIAA levels. The data, analyzed by two-way ANOVA, showed significant effects of diet (F = 64.6, df = 1, 30, *p* < 0.01), honey protein treatment (F = 89.4, df = 2, 30, *p* < 0.01), and the diet × honey protein treatment interaction (F = 21.9, df = 2, 30, *p* < 0.01). Post hoc analysis showed that 5-HIAA levels increased following honey protein administration in both the HFD and ND animals. HPD produced a significantly greater increase in 5-HIAA levels than LPD in the HFD rats. In the ND animals, both doses significantly increased 5-HIAA levels.

## 4. Discussion

The central serotonin and peripheral leptin systems present a complex interplay in the pathophysiology of obesity and its associated anxiety. Several studies have shown that chronic HFD consumption leads to hyperleptinemia, dyslipidemia, and impaired serotonergic signaling, manifesting as reduced locomotor activity and increased anxiety-like behavior [40,41,42].

The present study demonstrates that both honey and its glycoprotein components moderate the metabolic and neurochemical disturbances induced by HFD in rats. Our findings align with the reported literature linking obesity, leptin elevation, and central serotonin deficiency [36,43]. The HFD-fed rats exhibited significant weight gain (Figure 2), elevated serum triglycerides (Figure 5), and increased circulating leptin levels (Figure 6), a hallmark of metabolic inefficiency in obesity [44]. Treatment with HHD, HPD, and LPD resulted in decreased weight gain, lower circulating TAG, and attenuated hyperleptinemia, which may reflect improvements in lipid metabolism and energy homeostasis [32].

Furthermore, HFD-induced obesity was associated with hypoactivity in the open-field test (Figure 4), increased anxiety-like behavior (Figure 3), and reduced central serotonergic tone (Figure 7 and Figure 8), consistent with previous reports [36,45]. These findings demonstrate significant diet-dependent effects on metabolic, neurochemical, and behavioral outcomes, suggesting an integrated metabolic–CNS dysfunction [46]. Notably, by lowering serum leptin, TAG, weight gain, and anxiety-like behavior, and by improving central serotonin levels and locomotor activity, HHD partially reversed HFD-induced pathology, suggesting restoration of leptin–serotonin crosstalk.

These data highlight leptin–serotonin interactions as a pivotal therapeutic target, with dietary status dictating intervention efficacy. HPD yielded partial anxiolytic effects. Several agents have been reported to modulate 5-HT neurotransmission, including escitalopram [47]. Herein, HHD treatment significantly restored 5-HT and its metabolite 5-HIAA in both the ND and HFD rats, whereas LHD increased 5-HT only in the HFD rats. This suggests that honey may enhance tryptophan availability or TPH2 enzyme activity, resulting in increased serotonin synthesis [32]. In contrast, honey proteins primarily elevated 5-HIAA (*p* < 0.01 in the HFD rats) with minimal effects on 5-HT, suggesting selective augmentation of serotonin turnover rather than synthesis, possibly via MAO inhibition [48]. The antioxidant and anti-inflammatory properties of honey likely mitigate oxidative stress in hypothalamic neurons, thereby restoring leptin receptor sensitivity and TPH2 function [49,50,51]. The major royal jelly proteins (MRJPs) in honey exert immunomodulatory and lipid-catabolic effects [35], with low doses uniquely producing anxiolytic effects via serotonergic turnover [52,53,54,55].

Our data suggest a key interaction between leptin elevation and serotonergic dysfunction. Hyperleptinemia correlated inversely with 5-HT and 5-HIAA levels (*p* < 0.001), supporting a model in which leptin elevation impairs hypothalamic STAT3 signaling and reduces TPH2-mediated serotonin synthesis [56,57]. Honey likely restored this axis by improving leptin sensitivity and directly enhancing 5-HT production, whereas honey proteins primarily optimized serotonin catabolism independently of leptin signaling.

It should be noted, however, that the mechanisms involving leptin sensitivity, STAT3 signaling, TPH2-mediated serotonin synthesis, MAO activity, and leptin–serotonin crosstalk were not directly measured in the present study. Based on our findings, we propose that honey may exert its effects by improving leptin clearance or receptor sensitivity, while honey glycoproteins may modulate lipid metabolism via TAG lipolysis pathways. Furthermore, since 5-HT and 5-HIAA were measured in whole-brain homogenates, the observed changes reflect global serotonergic tone and cannot identify region-specific alterations or distinguish between synthesis and degradation mechanisms. These potential mechanisms should be explored in future studies to provide molecular-based evidence.

## 5. Conclusions

This study demonstrates that both natural honey and its glycoproteins (MRJPs) attenuate HFD-induced metabolic and neurobehavioral dysregulation in rats, via two distinct mechanisms. First, HHD lowered HFD-induced hyperleptinemia and enhanced central serotonin synthesis, potentially restoring metabolic homeostasis and reducing anxiety-like behavior. Second, honey proteins promoted serotonin turnover and reduced hyperleptinemia, offering distinct benefits for anxiety-like behavior. Natural honey appears effective in addressing anxiety-related phenotypes via enhanced 5-HT synthesis, whereas honey proteins may exert anxiolytic effects via increased 5-HIAA turnover.

These findings underscore the potential of honey-based interventions as multifaceted therapies for obesity-related metabolic and behavioral comorbidities. Future studies should validate these combinatorial approaches in clinical trials.

## Figures and Tables

**Table 1:** Groups of rats according to the intervention received.

Group No.	ND Groups	Group No.	HFD Groups
1	ND saline	6	HFD saline
2	ND low honey dose (LHD)	7	HFD low honey dose (LHD)
3	ND high honey dose (HHD)	8	HFD high honey dose (HHD)
4	ND low honey protein dose (LPD)	9	HFD low honey protein dose (LPD)
5	ND high honey protein dose (HPD)	10	HFD high honey protein dose (HPD)

## Data Availability

The original contributions presented in this study are included in the article/Supplementary Materials. Further inquiries can be directed to the corresponding authors.

## Supplementary Materials

The following supporting information can be downloaded at: https://www.mdpi.com/article/10.3390/biomedicines14071641/s1, Figure S1: Schematic presentation of the study. Grouping of the animals on the basis of weekly treatment; Figure S2: Different independent and dependent variables used in this study. The bottom row of boxes indicates methods used for quantifying these variables; Figure S3: (A) The caloric intake of ND and HFD treated with honey (HHD and LHD) in rats. Values are means ± SD. (B) ND and HFD rats treated with honey proteins (HPD and LPD). Values are means ± SD. (C) The caloric intake of ND and HFD treated with honey proteins (HPD and LPD) in rats.

## Abbreviations

HFD	High Fat Diet
ND	Normal Diet
EPM	Elevated plus maze
MRJP	Major royal jelly protein
HP	Honey proteins
HHD	High honey dose
LHD	Low honey dose
HPD	High protein dose
LPD	Low protein dose
5-HT	5-Hydroxytryptamine
5-HIAA	5-Hydroxyindoleacetic acid
