# Supplementary material for: Modulation of Leptin and Serotonin by Honey and Its Glycoproteins Against High-Fat Diet-Induced Metabolic and Anxiety Phenotypes
Source: Biomedicines. 2026 Jul 21;14(7):1641. doi: 10.3390/biomedicines14071641 (PMC13406404; doi:10.3390/biomedicines14071641)
Supplement: Supplementary file 1 [file biomedicines-14-01641-s001.zip › biomedicines-4299903-supplementary.pdf]

Supplementary Figures

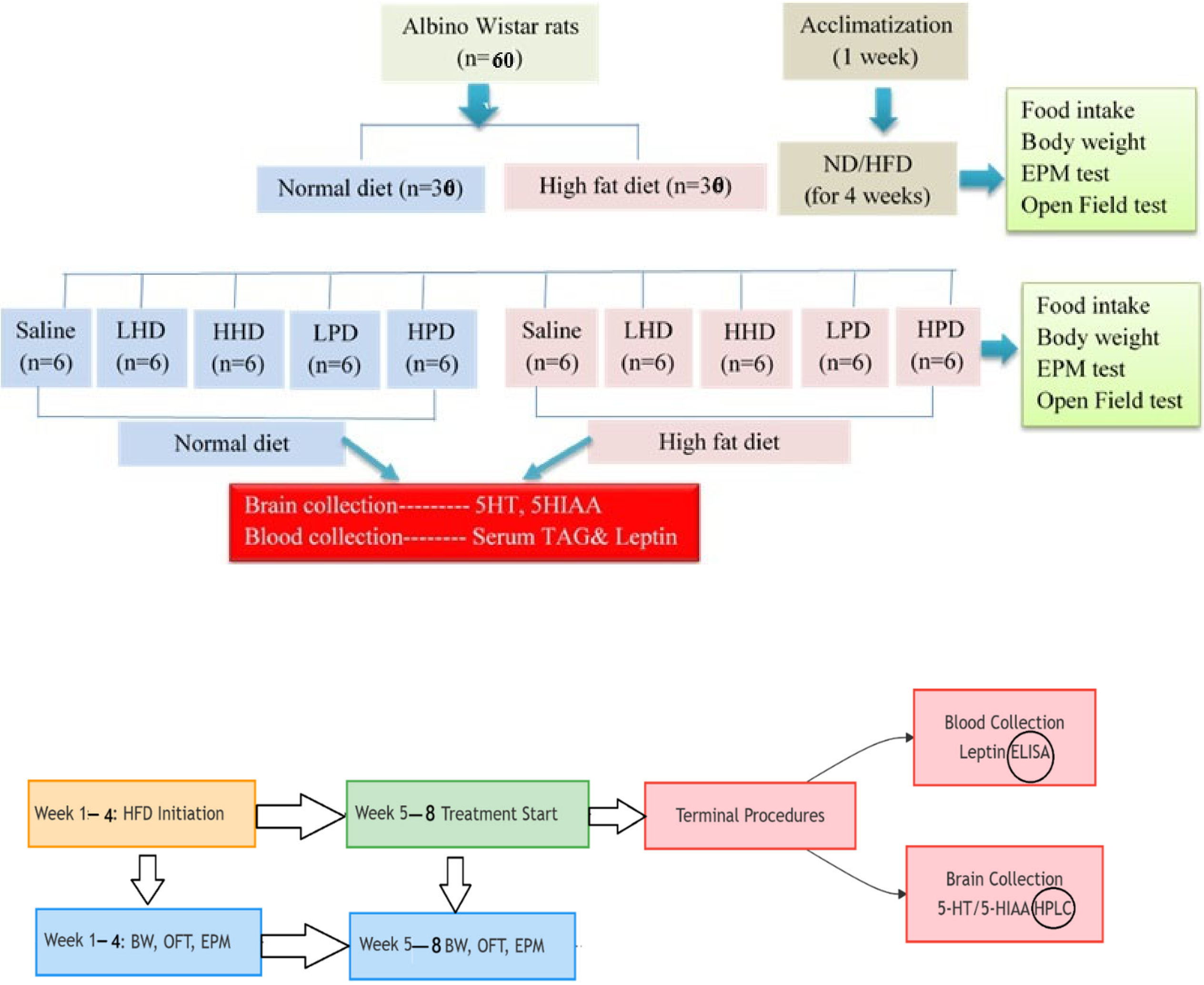

Figure S1: Schematic presentation of the study. Grouping of the animals on the basis of weekly treatment.

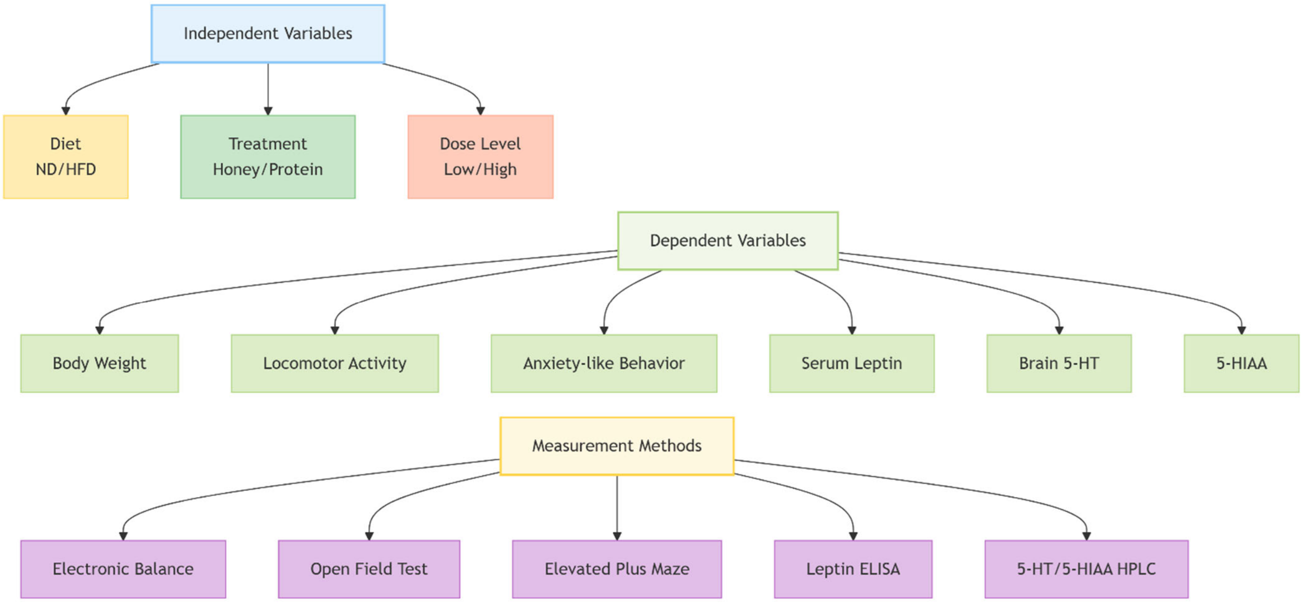

Figure S2: Different independent and dependent variables used in this study. The bottom row of boxes indicates methods used for quantifying these variables.

Caloric Intake

Caloric intakes for the two groups (ND and HFD without a treatment) of rats were calculated based on the energy content of each diet consumed by the rats. This analysis showed the difference in calories consumed by the HFD and the ND rats was non-significant ($F=0.45$, df = 1, 34 $p>0.05$) (Figure S3A). The effect of repeated measures was not significant ($F=533.9$, df = 4,136 $p>0.05$). Interaction between repeated measures x HFD ($F=0.59$, df = 4, 136, $p>0.05$) was also not significant. Post hoc test showed that there were no significant differences in caloric intake between the two groups.

Figure S3B shows the caloric intake of ND and HFD animals treated with honey (HHD and LHD). Three-way ANOVA (repeated measures design) revealed a significant effect of HFD [$F(1,30)=9.7$, $p<0.01$]. The effects of honey treatment [$F(2,30)=1.6$, $p>0.05$] and repeated measures [$F(4,120)=0.9$, $p>0.05$] on caloric intake were not significant. The interactions of repeated measures × HFD [$F(4,120)=0.5$, $p>0.05$], repeated measures × honey [$F(8,120)=0.1$, $p>0.05$], HFD × honey [$F(2,30)=1.8$, $p>0.05$], and repeated measures × HFD × honey [$F(8,120)=0.4$, $p>0.05$] were also not significant. Post hoc analysis showed no significant effects of HHD or LHD on caloric intake in HFD-treated animals compared with HFD saline controls.

Figure S3C shows the caloric intake in the experimental animals. The three-way ANOVA (repeated measure design) revealed that the HFD ($F=15.4$ df=1, N=30, $p<0.01$), honey proteins treatment ($F=7.6$, df=2, N=30, $p<0.01$), and repeated measures ($F=442.5$, df=4, 120, $p<0.01$) had significant effects on calorie intake. There was a significant interaction between weeks x HFD ($F=3.9$, df=4, 120, $p<0.05$) and weeks x honey proteins ($F=4.9$, df 8, 120, $p<0.05$). There was also a significant interaction between HFD x honey proteins ($F=4.2$, df=2, N=30, $p<0.05$) and weeks x HFD x honey ($F=2.4$, df 8, 120, $p<0.05$). Post hoc analysis showed that there were slight significant effects of LPD and HPD on caloric intake in the HFD and the ND treated animals on week 2 and 3.

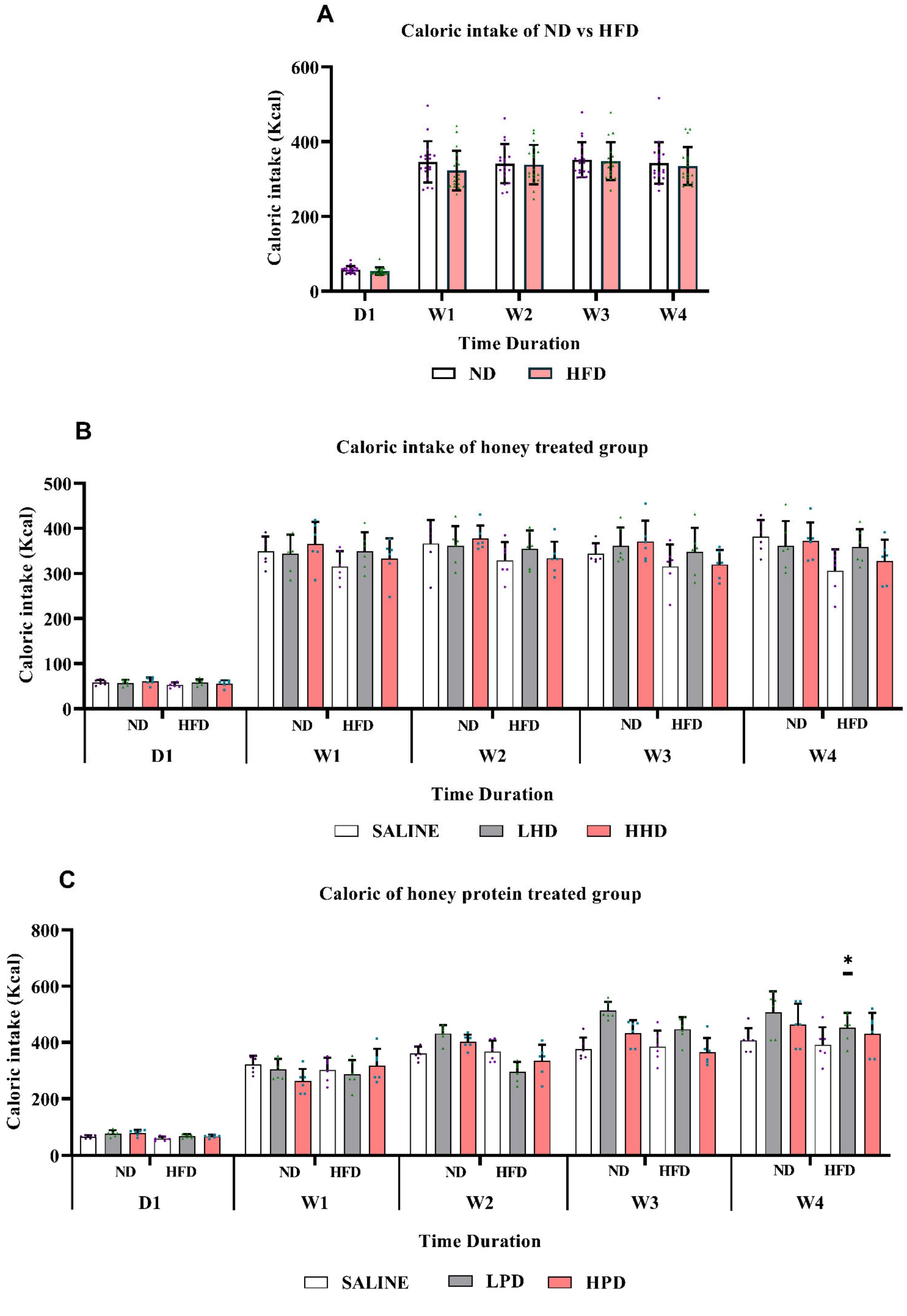

Figure S3: (A) The caloric intake of ND and HFD treated with honey (HHD and LHD) in rats. Values are means ± SD. (**B)** ND and HFD rats treated with honey proteins (HPD and LPD). Values are means ± SD. **(C)** The caloric intake of ND and HFD treated with honey proteins

(HPD and LPD) in rats. Values are means ± SD. Significant differences by Tukey's test: *p<0.01 from respective normal diet animals, +p<0.05, from HFD saline animals, $p<0.05 from respective LPD animals following three-way ANOVA (repeated measure design).
